# Biofeedback for training balance and mobility tasks in older populations: a systematic review

**DOI:** 10.1186/1743-0003-7-58

**Published:** 2010-12-09

**Authors:** Agnes Zijlstra, Martina Mancini, Lorenzo Chiari, Wiebren Zijlstra

**Affiliations:** 1 Center for Human Movement Sciences, University Medical Center Groningen, University of Groningen, Groningen, The Netherlands; 2 Department of Electronics, Computer Science & Systems, University of Bologna, Italy

## Abstract

**Context:**

An effective application of biofeedback for interventions in older adults with balance and mobility disorders may be compromised due to co-morbidity.

**Objective:**

To evaluate the feasibility and the effectiveness of biofeedback-based training of balance and/or mobility in older adults.

**Data Sources:**

PubMed (1950-2009), EMBASE (1988-2009), Web of Science (1945-2009), the Cochrane Controlled Trials Register (1960-2009), CINAHL (1982-2009) and PsycINFO (1840-2009). The search strategy was composed of terms referring to biofeedback, balance or mobility, and older adults. Additional studies were identified by scanning reference lists.

**Study Selection:**

For evaluating effectiveness, 2 reviewers independently screened papers and included controlled studies in older adults (i.e. mean age equal to or greater than 60 years) if they applied biofeedback during repeated practice sessions, and if they used at least one objective outcome measure of a balance or mobility task.

**Data Extraction:**

Rating of study quality, with use of the Physiotherapy Evidence Database rating scale (PEDro scale), was performed independently by the 2 reviewers. Indications for (non)effectiveness were identified if 2 or more similar studies reported a (non)significant effect for the same type of outcome. Effect sizes were calculated.

**Results and Conclusions:**

Although most available studies did not systematically evaluate feasibility aspects, reports of high participation rates, low drop-out rates, absence of adverse events and positive training experiences suggest that biofeedback methods can be applied in older adults. Effectiveness was evaluated based on 21 studies, mostly of moderate quality. An indication for effectiveness of visual feedback-based training of balance in (frail) older adults was identified for postural sway, weight-shifting and reaction time in standing, and for the Berg Balance Scale. Indications for added effectiveness of applying biofeedback during training of balance, gait, or sit-to-stand transfers in older patients post-stroke were identified for training-specific aspects. The same applies for auditory feedback-based training of gait in older patients with lower-limb surgery.

**Implications:**

Further appropriate studies are needed in different populations of older adults to be able to make definitive statements regarding the (long-term) added effectiveness, particularly on measures of functioning.

## Introduction

The safe performance of balance- and mobility-related activities during daily life, such as standing while performing manual tasks, rising from a chair and walking, requires adequate balance control mechanisms. One-third to one-half of the population over age 65 reports some difficulty with balance or ambulation [1]. The disorders in balance control can be a consequence of pathologies, such as neurological disease, stroke, diabetes disease or a specific vestibular deficit, or can be due to age-related processes, such as a decline in muscle strength [2,3], sensory functioning [4], or in generating appropriate sensorimotor responses [5]. Balance and mobility disorders can have serious consequences regarding physical functioning (e.g. reduced ability to perform activities of daily living) as well as psycho-social functioning (e.g. activity avoidance, social isolation, fear of falls) and may even lead to fall-related injuries.

Because of the high incidence of balance and mobility disorders in older adults and the large negative impact for the individual, interventions are necessary that optimize the performance of balance- and mobility-related activities in specific target populations of older adults. Beneficial effects of balance- and mobility-related exercise interventions have been demonstrated, for example, in healthy and frail older adults [6]. Providing individuals with additional sensory information on their own motion, i.e. biofeedback, during training may enhance movement performance. Depending on the functioning of the natural senses that contribute to balance control, i.e. the vestibular, somatosensory, and visual systems [7], the biofeedback may be used as a substitute [8] or as an augmentation [9] in the central nervous system's sensorimotor integration. Enhanced effects on movement performance after training with augmented biofeedback may be caused by 'sensory re-weighing' processes, in which the relative dependence of the central nervous system on the different natural senses in integrating sensory information is modified [10,11].

The effects of biofeedback-assisted performance of balance and mobility tasks have been investigated in experimental studies [12-16]. Whether biofeedback-based training is effective for improving movement performance after an intervention has been systematically analyzed for stroke rehabilitation [17-19]. Despite the possible relevance for supporting independent functioning in older adults, thorough investigations on the effectiveness of biofeedback-based interventions for training balance and mobility in different populations of older adults have not been conducted yet. Hence, there is limited evidence so far on whether the successful application of biofeedback-based interventions could be compromised in older adults with balance or mobility disorders due to the existence of co-morbidity. Besides disabling health conditions, such as musculoskeletal impairments and cardiovascular problems, declines in sensory functioning and/or cognitive capabilities can exist in persons of older age. Since the possibility of disabling health conditions and difficulties in the processing of biofeedback signals, there is a need for evaluations of interventions that apply biofeedback for improving balance and mobility in older adults. Therefore, the objectives of the present systematic review are to evaluate the feasibility and the effectiveness of biofeedback-based interventions in populations of healthy older persons, mobility-impaired older adults as well as in frail older adults, i.e. older adults that are characterized by residential care, physical inactivity and/or falls.

## Methods

### Data sources and searches

Relevant studies were searched for in the electronic databases PubMed (1950-Present), EMBASE (1988-Present), Web of Science (1945-Present), the Cochrane Controlled Trials Register (1960-Present), CINAHL (1982-Present) and PsycINFO (1840-Present). The search was run on January 13th 2010. The following search strategy was applied in the PubMed database:

#1 **Biofeedback (Psychology) **OR (biofeedback OR bio-feedback OR "augmented feedback" OR "sensory feedback" OR "proprioceptive feedback" OR "sensory substitution" OR "vestibular substitution" OR "sensory augmentation" OR "auditory feedback" OR "audio feedback" OR audio-feedback OR "visual feedback" OR "audiovisual feedback" OR "audio-visual feedback" OR "somatosensory feedback" OR "tactile feedback" OR "vibrotactile feedback" OR "vibratory feedback" OR "tilt feedback" OR "postural feedback")

#2 **Movement **OR **Posture **OR **Musculoskeletal Equilibrium **OR (movement OR locomotion OR gait OR walking OR balance OR equilibrium OR posture OR postural OR sit-to-stand OR stand-to-sit OR "bed mobility" OR turning)

#3 **Middle Aged **OR **Aged **OR ("older people" OR "old people" OR "older adults" OR "old adults" OR "older persons" OR "old persons" OR "older subjects" OR "old subjects" OR aged OR elderly OR "middle-aged" OR "middle aged" OR "middle age" OR "middle-age")

#4 (1 AND 2 (AND 3))

in which the bold terms are MeSH (Medical Subjects Headings) key terms. The search strategy was formulated with assistance of an experienced librarian. Since the EMBASE, Web of Science, CINAHL and PsycINFO databases do not have a MeSH key terms registry, the depicted strategy was modified for these databases. To identify further studies, 'Related Articles' search in PubMed, and 'Cited Reference Search' in Web of Science was performed and reference lists of primary articles were scanned.

### Study selection

Different criteria were applied in selecting studies for evaluating (1) the feasibility, and (2) the effectiveness of biofeedback-based training programs for balance and/or mobility in older adults. Biofeedback was defined as measuring some aspect of human motion or EMG activity and providing the individual, in real-time, with feedback information on the measured signal through the senses. Mobility stands for any activity that results in a movement of the whole body from one position to another, such as in transfers between postures and walking.

#### • Study selection criteria - Feasibility of biofeedback-based interventions

All available intervention studies were considered that were published in the years 1990 up to 2010 and that applied biofeedback for repeated sessions of training balance and/or mobility tasks in older adults. Biofeedback studies that only evaluated one experimental session were excluded. No selection was made regarding the (non) use of a control-group design. The criterium of a mean age of 60 years or above for the relevant subject group(s) was applied for including studies in 'older adults'. No selection was made regarding the (non)existence of specific medical conditions.

#### • Study selection criteria - Effectiveness of biofeedback-based interventions

Studies that were published up to 2010 were considered for the effect evaluation. In addition to the criteria for selecting studies in evaluating the feasibility of biofeedback-based interventions, studies had to comply with the following criteria.

(1) Control-group design. Since the effect evaluation focused on the 'added effect' of applying biofeedback-based training methods, studies comparing biofeedback-based training to similar training without biofeedback or to conventional rehabilitation were considered. In addition, studies comparing a biofeedback-based training group to a control group of older adults that did not receive an exercise-based intervention were included. Non-controlled and case studies were excluded.

(2) Objective outcomes. Studies were considered if they used at least one objective measure of performing a balance or mobility task. Studies that only used measures of muscle force or EMG activity were excluded.

#### • Selection procedures

The titles and abstracts of the results obtained by the database search were screened by 2 independent reviewers (AZ & MM). The full-text articles of references that were potentially relevant were independently retrieved and examined. A third reviewer (WZ) resolved any discrepancies. Only full-text articles that were in English, Italian or Dutch were retrieved. In case a full-text article did not exist, the author was contacted to provide study details.

### Quality assessment

The quality of the selected studies in evaluating the effectiveness was rated with use of the PEDro scale (see table 1 for a description of the different items). The scale combines the 3-item Jadad scale and the 9-item Delphi list, which both have been developed by formal scale development techniques [20,21]. In addition, "fair" to "good" reliability (ICC = .68) of the PEDro scale for use in systematic reviews of physical therapy trials has been demonstrated [22]. The PEDro score, which is a total score for the internal and statistical validity of a trial, was obtained by adding the scores on items 2-11. A total score for the external validity was obtained by adding the score on item 1 of the PEDro scale and the score on an additional item (see table 1 item 12), that was derived from a checklist by Downs & Black [23]. One point was awarded if a criterion was satisfied on a literal reading of the study report. Two reviewers (AZ & MM) independently scored the methodological quality of the selected studies and a third reviewer (WZ) resolved any disagreements.

**Table 1:** Criteria that were used in rating the methodological quality of relevant studies.

	Criteria of the PEDro scale:
	*External validity*
1	Eligibility criteria were specified.
	*Internal and statistical validity*
2	Subjects were randomly allocated to groups.
3	Allocation was concealed.
4	The groups were similar at baseline regarding the most important prognostic indicators.
5	There was blinding of all subjects.
6	There was blinding of all therapists who administered the therapy.
7	There was blinding of all assessors who measured at least one key outcome.
8	Measurements of at least one key outcome were obtained from more than 85% of the subjects initially allocated to groups.
9	All subjects for whom outcome measurements were available received the treatment or control condition as allocated, or where this was not the case, data for at least one key outcome were analyzed by "intention to treat".
10	The results of between-group statistical comparisons are reported for at least one key outcome.
11	The study provides both point measurements and measurements of variability for at least one key outcome.

	**Additional criterion external validity:**
12	The staff, places and facilities where the patients were treated, were representative of the staff, places and facilities where the majority of the patients are intended to receive the treatment.

### Analysis of relevant studies

Studies that complied with the selection criteria for evaluating the feasibility of biofeedback-based interventions in older adults or for the effect evaluation were categorized into groups. A group consisted of at least 2 studies that evaluated similar type of interventions, or that had similar training goals, and that were in similar types of older participants.

#### • Feasibility of biofeedback-based interventions

Information on the following aspects were extracted from the articles: (1) adherence to the training program, (2) occurrence of adverse events, (3) exclusion of subjects with co-morbidity, (4) usability of the biofeedback method in understanding the concept of training and in performing the training tasks, (5) attention load and processing of the biofeedback signals, (6) subject's acceptance of the biofeedback technology, and (7) subject's experience and motivation during training. Information on adherence to the biofeedback-based training program was collected by extracting participation rates and information on drop-outs.

#### • Effectiveness of biofeedback-based interventions

A standardized form was developed to extract relevant information from the included articles. A first version was piloted on a subset of studies and modified accordingly. As outcomes, objective measures for quantifying an aspect of performing a balance or mobility task were considered. In addition, self-report or observation of functional balance or mobility, motor function, ability to perform activities of daily living, level of physical activity, and the number of falls during a follow-up period were considered. Effect sizes were calculated for outcomes for which a significant between-group difference was reported in favor of the experimental group, i.e. the group of subjects that had received training with biofeedback. Pre- to post-intervention effect sizes were calculated by subtracting the difference in mean scores for the control group from the difference in mean scores for the experimental group and dividing by the control-group pooled standard deviation of pre, post values [24]. Interpretation of the effect size calculations were consistent with the categories presented by Cohen [25]: small (< 0.41), moderate (0.41 to 0.70), and large (> 0.70). A qualitative analysis was performed in which occurrences of (non)significant effects for the same type of outcome in 2 or more similar studies were identified. After an initial screening of the literature search results, it was decided to perform a qualitative analysis, since the amount of relevant studies and the similarity in outcome measures and testing procedures was considered insufficient to perform a solid quantitative analysis.

## Results

In total, 27 studies [26-55] (publication years 1990-2009) were selected for evaluating feasibility of biofeedback-based interventions. The 2 articles by Sihvonen et al [48,49] report on the same study. Also, the articles by Eser et al [34] and Yavuzer et al [55] as well as the 2 articles by Engardt (et al) [32,33] report on the same study. For evaluating effectiveness of biofeedback-based interventions, 21 controlled studies [26,28-30,32,33,35,38-42,44-49,51,52,55-57] (all publication years up to and including 2009) were considered. A full description of the selection process and search results is given in a next section. The patients included in the study of Grant et al [35] were a subset of the study of Walker et al [51]. The study of Grant et al [35] was therefore used for outcomes not investigated by Walker et al [51].

### Feasibility of biofeedback-based interventions

#### • Training balance with visual biofeedback in (frail) older adults

Five [31,46,48,49,52,53] out of 14 studies [27,31,36-39,42,43,46,48-50,52-54] included persons with debilitating conditions such as indicated by residential care, falls or inactivity. Five studies reported on aspects of feasibility. Lindemann et al [43] mentioned that there was no occurrence of negative side effects during 16 sessions of training balance on an unstable surface in 12 older adults. Wolfson et al [54], who combined biofeedback and non-biofeedback training, reported that the attendance at the sessions was 74% while 99% of the subjects was able to participate in all of the exercises. Wolf et al [53] reported that 4 out of 64 older adults dropped out of a 15-week intervention for training balance on movable pylons due to prolonged, serious illness or need to care for an ill spouse. In a study by De Bruin et al [31] 4 out of 30 subjects dropped out of a 5-week intervention due to medical complications that interfered with training. The remaining subjects were all able to perform the exercises on a stable and unstable platform and complied with 94% of the scheduled training sessions. Sihvonen et al [48,49] mentioned that no complications had occurred during a 4-week intervention in 20 frail older women and that the participation rate was 98%. Furthermore, they mentioned that the training method and the exercises could easily be adapted to the health limitations of the older women.

#### • Training balance with visual biofeedback in older patients post-stroke

In general, the patients in the 5 available studies [30,34,35,47,51,55] were without co-morbidity, impaired vision or cognition. Two studies reported on aspects of feasibility. In the study described by Yavuzer et al [55] and Eser et al [34], none of the patients missed more than 2 therapy sessions. Three out of 25 patients dropped out of a 3-week intervention due to early discharge from the clinic for non-medical reasons. Sackley & Lincoln [47] reported that 1 out of 13 patients dropped out of a 4-week intervention due to medical complications. The patients commented that they enjoyed the biofeedback treatment because they knew exactly what they were required to achieve and could judge the results for themselves. Furthermore, patients with quite severe communication problems found the visual information easy to understand and grasped the concept of training more effectively than with conventional treatment.

#### • Training gait with auditory (and visual [28]) biofeedback in older patients post-stroke

In general, the patients in the 4 available studies [26,28,44,45] did not have additional neurological conditions or malfunction of the leg(s). Bradley et al [28] mentioned that all but one patient performed all 18 training sessions and that 1 out of 12 patients stopped participation due to full recovery.

#### • Training sit-to-stand transfers with auditory [32,33] or visual [29] biofeedback in older patients post-stroke

In both available studies [29,32,33], patients did not have severe cognitive deficits and in the study by Cheng et al [29] patients did not have additional neurological conditions and did not have arthritis or fractures in the lower extremities. Engardt et al [32,33] mentioned that 1 out of 21 patients dropped out of a 6-week intervention and that patients focused more on initiating the audio-signal, which indicated sufficient weight-bearing on the paretic leg, than on rising up.

#### • Training gait with auditory biofeedback in older patients with lower-limb surgery

Hershko et al [40] excluded patients with major cognitive impairment, fractures or operations in the opposite lower limb or with neurological disease. Isakov et al [41] did not mention patient exclusion criteria. Both available studies did not report on aspects of feasibility.

### Effectiveness of biofeedback-based interventions - Search results

**Figure 1:**
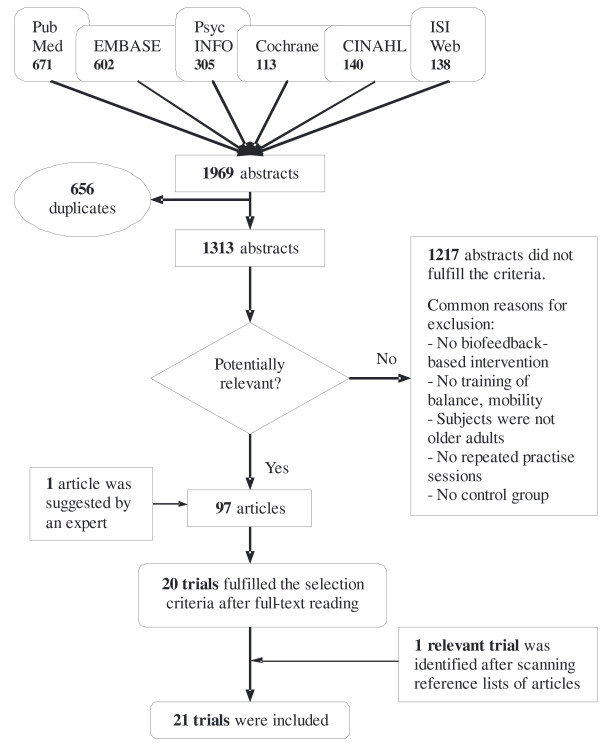
**Study selection procedure for evaluating effectiveness of biofeedback-based interventions**. At the top of the figure, the utilised literature databases are shown.

A flow diagram of the search and selection process is depicted in figure 1. A number of biofeedback studies, on repeated practice of balance and/or mobility tasks in older adults, that included a comparison group were nevertheless excluded. An overview of the excluded studies is given in table 2. The descriptive characteristics of the 21 included studies are summarized in table 3. Seventeen studies were randomized controlled trials. The number of subjects in the experimental group was small to moderate, i.e. varying from 5-30 subjects. Six studies included (frail) older adults that did not have a specific medical condition, but for example had a history of falls or were physically inactive. Twelve studies included older patients post-stroke and 3 studies included older patients with lower-limb surgery, i.e. below- or above-knee amputation, hip or knee replacement, femoral neck fracture, hip nailing, tibial plateau or acetabular surgery.

**Table 2:** Studies excluded for evaluating effectiveness of biofeedback-based interventions.

Authors	Reason for exclusion
Bisson et al [27]	Comparison of BF vs. virtual reality training
Burnside et al [62]	No objective measure of a balance/mobility task
De Bruin et al [31]	Comparison of 2 different forms of BF training
Eser et al [34]	No objective measure of a balance/mobility task
Gapsis et al [63]	No objective measure of a balance/mobility task
Hamman et al [36]	No control group with older adults
Hatzitaki et al [37]	Pre-, post-testing in a moving obstacle avoidance task
Lindemann et al [43]	BF training was compared to home-based exercise
Mudie et al [64]	Training of sitting balance
Santilli et al [65]	No objective measure of a balance/mobility task
Ustinova et al [50]	No control group with older adults
Wissel et al [66]	No objective measure of a balance/mobility task
Wolf et al [53]	No objective measure of a balance/mobility task
Wolfson et al [54]	Comparison does not allow for evaluating BF-part
Wu [67]	Only 2 control subjects, no comparison of group means

**Table 3:** Characteristics of included studies for evaluating effectiveness of biofeedback-based interventions.

A. Visual biofeedback-based training of balance in (frail) older adults							
**Reference Location**	**Design**	**Population** **Mean age (years)**	**Group size Drop-outs**	**Equipment**	**Biofeedback type,** **comparison group(s)**	**Frequency Duration**^**a**^	**Short-term outcomes**

Hatzitaki et al[38] 2009 Greece	RCT	Community-dwelling, older women E1 = 71, E2 = 71^b ^C = 71	E1 = 19, E2 = 15^b ^C = 14	ERBE Balance System: force plate system with display	Continuous visual feedback of force vector under each foot vs no intervention	3× wk, 4 wks 25 minutes Total: 300 min	COP asymmetry during standing, sway during normal and tandem standing.
Heiden & Lajoie[39] 2009 Canada	CT	Community-dwelling, older adults recruited from a chair exercise program 77	E = 9, C = 7	NeuroGym Trainer: games- based system with 2 pressure sensors & display	Visual feedback of the difference in signal between the 2 sensors in controlling a virtual tennis game vs no intervention, both in addition to a chair exercise program	2× wk, 8 wks 30 minutes Total: 480 min	Sway and RT during standing with feet together. CB&M scale, 6-minute walk distance
Lajoie[42] 2004 Canada	CT	Older adults from residential care facilities or living in the community E = 70, C = 71	E = 12, C = 12	Force plate system with display	Continuous visual feedback of COP (feedback-fading protocol) vs no intervention	2× wk, 8 wks 60 minutes Total: 960 min	Sway and RT during standing with feet together. BBS
Rose & Clark[46] 2000 USA	CT	Older adults with a history of falls 79	E = 24, C = 21	Pro Balance Master system: force plate system with display	Continuous visual feedback of COG (feedback-fading protocol) vs no intervention	2× wk, 8 wks 45 minutes Total: 720 min	Sway (SOT) and weight-shifting (100%LOS) during standing. BBS, TUG
Sihvonen et al[48,49] 2004 Finland	RCT	Frail older women living in residential care homes E = 81, C = 83	E = 20, C = 8 1 C	Good Balance system: force plate system with display	Continuous visual feedback of COP vs no intervention	3× wk, 4 wks 20-30 minutes Total: 240-360 min	Sway during standing, varying vision and base of support & weight-shifting during standing. BBS, activity level
Wolf et al[52] 1997 USA	RCT	Physically inactive older adults from independent-living center E = 78, C1 = 78, C2 = 75	E = 24, C1 = 24 C2 = 24	Chattecx Balance System: force plate system with display	Continuous visual feedback of COP vs Tai Chi chuan training vs Educational sessions	1× wk, 15 wks 60 minutes Total: 900 min	Sway during standing, varying vision and base of support.

**B. Visual biofeedback-based training of balance in older patients post-stroke**							

**Reference Location**	**Design**	**Population** **Mean age (years)**	**Group size Drop-outs**	**Equipment**	**Biofeedback type,** **comparison group(s)**	**Frequency Duration^a^**	**Short-term outcomes**

Cheng et al[30] 2004 Taiwan	CT	Patients post-stroke E = 61, C = 61	E = 30, C = 25 2 E, 1 C	Balance Master: force plate system with display	Continuous visual feedback of COG & conv. therapy vs conv. therapy	5× wk, 3 wks 20 minutes Total: 300 min	Sway during standing, varying vision and surface movement & weight-shifting during standing
*Grant et al*[35] 1997 Canada	RCT	Patients post-stroke 65	E = 8, C = 8 1	Balance Master: force plate system with display	Continuous visual feedback of COG vs conv. balance training, both in in addition to conv. therapy	2 to 5× wk, max. 8 wks 30 minutes Total: 570 min (average)	Weight-distribution during standing
*Sackley & Lincoln*[47] 1997 UK	RCT	Patients post-stroke E = 61, C = 68	E = 13, C = 13 1 E	Nottingham Balance Platform: force plate system with display	Continuous visual feedback of weight on the legs vs same training without feedback, both as part of functional therapy and in addition to conv. therapy	3× wk, 4 wks 20 minutes Total: 240 min	Sway and weight-distribution during standing. RMA, Nottingham ADL scale
*Shumway et al*[57] 1988 USA	RCT	Patients post-stroke E = 66, C = 64	E = 8, C = 8	Force plate system with display	Continuous visual feedback of COP vs conv. balance training, both as part of conv. therapy	2× day, 2 wks 15 minutes Total: 300 min	Sway and weight-distribution during standing
*Walker et al*[51] 2000 Canada	RCT	Patients post-stroke E = 65, C1 = 62, C2 = 66	E = 18, C1 = 18 C2 = 18 2 E, 2 C1, 4 C2	Balance Master: force plate system with display	Continuous visual feedback of COG and weight on the legs vs conv. balance training, both in addition to conv. therapy vs conv. therapy	5× wk, 3-8 wks 30 minutes Total: 450-1200 min	Sway during standing, varying vision. BBS, TUG, max. gait velocity test
Yavuzer et al[55] 2006 Turkey	RCT	Patients post-stroke E = 60, C = 62	E = 25, C = 25 3 E, 6 C	Nor-Am Target Balance Training System: portable force plate system with display	Continuous visual feedback of COG & conv. therapy vs conv. therapy	5× wk, 3 wks 15 minutes Total: 225 min	Gait: time-distance, kinematic and kinetic parameters

**C. Auditory (& visual) biofeedback-based training of gait in older patients post-stroke**							

**Reference Location**	**Design**	**Population** **Mean age (years)**	**Group size** **Drop-outs**	**Equipment**	**Biofeedback type,** **comparison group(s)**	**Frequency Duration^a^**	**Short-term outcomes**

*Aruin et al*[26] 2003 USA	RCT	Patients post-stroke and narrow base of support during walking 65	E = 8, C = 8	2 sensors placed below knees and next to tibial tuberosity & wearable unit providing signals	Auditory feedback of distance between knees during conv. therapy vs conv. therapy	2× day, 10 days 25 minutes Total: 500 min	Step width during walking
*Bradley et al*[28] 1998 UK	RCT	Patients post-stroke E1 = 67, E2 = 72, C1 = 77, C2 = 68^c^	E1 = 5, E2 = 7 C1 = 5, C2 = 6^c^ 2 C1	Portable EMG device	Auditory & visual feedback of muscle tone during conv. therapy vs conv. therapy	18×, 6 wks ? minutes	Step length, stride width, foot angle during walking & RMI & Nottingham Extended ADL Index
*Montoya et al*[44] 1994 France	RCT	Patients post-stroke E = 64, C = 60	E = 9, C = 5	Walkway with lighted targets & locometer	Auditory feedback of step length vs same training without feedback, both in addition to conv. therapy	2× wk, 4 wks 45 minutes Total: 360 min	Step length of paretic side during walking
*Morris et al*[45] 1992 Australia	RCT	Patients post-stroke and knee hyperextension E = 64, C = 64	E = 13, C = 13	Electrogoniometric monitor	Auditory feedback of knee angle during conv. therapy (phase 1) vs conv. therapy (phase 1), both followed by conv. therapy (phase 2)	1× wk, 4 wks 30 minutes Total: 600 min	Velocity, asymmetry and peak knee extension during walking & MAS (gait)

**D. Visual or auditory biofeedback-based training of sit-to-stand transfers in older patients post-stroke**							

**Reference Location**	**Design**	**Population** **Mean age (years)**	**Group size Drop-outs**	**Equipment**	**Biofeedback type,** **comparison group(s)**	**Frequency Duration^a^**	**Short-term outcomes**

*Cheng et al*[29] 2001 Taiwan	RCT	Patients post-stroke E = 62, C = 63	E = 30, C = 24	Force plate system with voice instruction system, numerical LED and mirror	Visual feedback of weight-bearing symmetry, as part of conv. therapy vs conv. therapy	5× wk, 3 wks 50 minutes Total: 750 min	-, only long-term outcomes are reported
*Engardt et al*[32] 1993 Sweden	RCT	Patients post-stroke E = 65, C = 65	E = 21, C = 21 1 E, 1 C	Portable force-plate feedback system	Auditory feedback of weight on paretic leg vs same training without feedback, both in addition to conv. therapy	3× day, 6 wks 15 minutes Total: 1350 min	Weight-distribution during rising and siting down. BI (self-care & mobility), MAS (sit-stand)

**E. Auditory biofeedback-based training of weight-bearing during balance tasks **[56]** or gait tasks in older patients with lower-limb surgery**							

**Reference Location**	**Design**	**Population** **Mean age (years)**	**Group size Drop-outs**	**Equipment**	**Biofeedback type,** **comparison group(s)**	**Frequency Duration^a^**	**Short-term outcomes**

*Gauthier et al*[56] 1986 Canada	RCT	Unilateral below-knee amputees E = 60, C = 65	E = 5, C = 6	Limb Load Monitor: Pressure sensitive sole	Auditory feedback of weight on prosthesis during conv. therapy vs conv. therapy	1× day, 8 days 10 minutes Total: 80 min	Sway and weight-distribution during standing, varying vision
*Hershko et al*[40] 2008 Israel	RCT	Patients with unilateral hip, tibial plateau or acetabular surgery 68	E1 = 9, E2 = 6 C1 = 8, C2 = 10^d^	SmartStep: in-shoe sole	Auditory feedback of weight on affected leg during PWB therapy vs PWB therapy, both followed by by conv. therapy	1× day, 5 days 35 minutes Total: 175 min	PWB on injured leg during walking & TUG
*Isakov*[41] 2007 Israel	RCT	Patients with below- or above-knee amputation, hip or knee replacement or femoral-neck fracture E = 62, C = 66	E = 24, C = 18	SmartStep: in-shoe sole	Auditory feedback of weight on affected leg during FWB therapy vs FWB therapy	2× wk, 2 wks 30 minutes Total: 120 min	FWB on injured leg during walking

References in italic represent the studies for which the added benefit of applying biofeedback could be evaluated.

^a ^Frequency and duration of biofeedback-based training only

^b ^Hatzitaki et al: subjects were divided into subgroups that practised weight-shifting in the anterior/posterior direction (E1) vs medio/lateral direction (E2)

^c ^Bradley et al: patients were divided into mild (C1, E1) and severe (C2, E2) subgroups according to their score on the RMI

^d ^Hershko et al: patients were instructed with Touch (= up to 20% of body weight, E1 & C1) or Partial (= 21-50% of body weight, E2 & C2) Weight-Bearing

### Effectiveness of biofeedback-based interventions - Quality assessment results

The initial, inter-rater agreement for the 2 reviewers was 76% in assessing external validity and 89% in assessing internal and statistical validity. This resulted in a total Cohen's Kappa score of 0.73, which is substantial (.61-.80) according to Landis and Koch's benchmarks for assessing the agreement between raters [58]. The main criteria on which disagreement occurred were representativeness of treatment staff, places and facilities; similarity of groups at baseline; and concealment of allocation.

In table 4 the total scores for methodological quality are reported. The eligibility criteria were specified by most authors, except for Cheng et al [29,30], Aruin et al [26], and Isakov [41]. The places and facilities where the experimental session took place were in most cases representative of the places and facilities where the majority of the target patients are intended to receive the treatment. However, in the study by Hatzitaki et al [38] and Rose & Clark [46], the experimental intervention was performed at a research laboratory. Furthermore, Aruin et al [26], Heiden & Lajoie [39], Montoya et al [44], Lajoie [42] and Wolf et al [52] did not mention where the training sessions took place.

**Table 4:** Quality scores and results of included studies for evaluating effectiveness of biofeedback-based interventions.

A. Visual biofeedback-based training of balance in (frail) older adults			
**Ref**	**Quality^a ^** **EV PEDro**	**Analysis^b ^Main short-term results**	**Effect sizes (absolute numbers)**

Hatzitaki et al[38]	1 | 5	rANOVA & post-hoc testing. Significant interactions between group and time, in favor of experimental group (E1^c^), for 2 of 4 asymmetry and 4 of 4 sway outcomes for tandem standing.	Asymmetry = 1.40 & 1.08 Sway = 0.38, 0.56, 0.69, 0.78
Heiden & Lajoie[39]	1 | 5	rANOVA & post-hoc testing. Significant interactions between group and time, in favor of experimental group, for RT and CB&M.	RT, CB&M = - (values are given in bar charts)
Lajoie[42]	1 | 4	rANOVA & post-hoc testing. Significant between-group differences for RT and BBS at posttest in favor of experimental group.	RT, BBS = - (values are given in bar charts)
Rose & Clark[46]	1 | 2	Doubly multivariate rANOVA & post-hoc testing. Significant interactions between group and time in favor of experimental group.	Sway = .51 Weight-shifting = .38 & .79 & .85 BBS = .46; TUG = .55
Sihvonen et al[48,49]	2 | 6	rANOVA & Friedman's test. Significant interactions between group and time, in favor of experimental group, for 2 of 6 weight-shifting, 4 of 18 sway outcomes and BBS. Significant improvement in activity level in experimental group.	Sway = .56 & .86 to 1.12 Weight-shifting = .77 & 1.29 BBS = .34 Activity level = - (categorical variable)
Wolf et al[52]	0 | 4	rANOVA with baseline characteristics and sway as covariates & post-hoc testing. Significant between-group differences in improvement for 5 of 12 sway outcomes in favor of experimental group.	Sway = .43 & .89 to 1.71

**B. Visual biofeedback-based training of balance in older patients post-stroke**			

**Ref**	**Quality^a^** **EV PEDro**	**Analysis^b^** **Main short-term results**	**Effect sizes (absolute numbers)**

Cheng et al[30]	1 | 4	rANOVA & post-hoc testing. Significant between-group differences in weight-shifting at posttest in favor of experimental group.	Weight-shifting = .59 & .78 to .90
*Grant et al*[35]	2 | 5	rANOVA & post-hoc testing. No significant between-group difference.	
*Sackley & Lincoln*[47]	2 | 6	Student's *t*-test & Mann-Whitney *U*-test. Significant between-group differences in weight- distribution, ADL and motor function at post-test in favor of experimental group.	Weight-distribution = .99 ADL = 1.21 Motor function = .99
*Shumway et al*[57]	2 | 4	Chi-square test. Significant between-group difference in change score for weight-distribution in favor of experimental group.	Weight-distribution = - (values are given in box plots)
*Walker et al*[51]	2 | 6	rANOVA & post-hoc testing. No significant between-group differences.	
Yavuzer et al[55]	2 | 6	Mann-Whitney *U*-test. Significant between-group differences in change scores for 2 of 17 gait outcomes in favor of experimental group.	Pelvic obliquity = .55^d^ Peak vGRF paretic side = .54

**C. Auditory (& visual **[28]**) biofeedback-based training of gait in older patients post-stroke**			

**Ref**	**Quality^a^** **EV PEDro**	**Analysis^b^** **Main short-term results**	**Effect sizes (absolute numbers)**

*Aruin et al*[26]	0 | 4	rANOVA Significant between-group difference after the intervention in favor of experimental group.	Step width = - (mean (SE) are given: .09 m(.003) to .16 m(.006) *vs*. 10 m(.004) to .13 m(.003))
*Bradley et al*[28]	2 | 5	Mixed model rANOVA (sign. if ?). No significant between-group differences.	
*Montoya et al*[44]	1 | 3	Factorial rANOVA^e^. Significant between-group difference, interaction between beginning/end and group, interaction between session and group, all in favor of experimental group.	Step length = 3.33
*Morris et al*[45]	2 | 7	Mann-Whitney *U*-test. Significant between-group difference for reduction in peak knee extension during phase 2 in favor of experimental group.	Peak knee extension = **-** (mean reduction (SD) are given: 1.7°(1.8) *vs*. 4°(3.1) (phase 2))

**D. Visual **[29]**or auditory biofeedback-based training of sit-to-stand transfers in older patients post-stroke**			

**Ref**	**Quality^a^** **EV PEDro**	**Analysis^b^Main short-term results**	**Effect sizes (absolute numbers)**

*Cheng et al*[29]	1 | 5	- (only long-term results)	- (only long-term results)
*Engardt et al*[32]	2 | 5	Student's *t*-test (sign. if *p *< .01) & Mann-Whitney *U*-test. Significant between-group differences in improvement for weight-distribution and functional sit-to-stand in favor of experimental group.	Weight-distribution = 1.16 & 1.47 Functional sit-to-stand = **-** (median (range) are given: 2(2) to 6(2-6) *vs *2(2) to 4(2-6))

**E. Auditory biofeedback-based training of weight-bearing during balance tasks **[56]**or gait tasks in older patients with lower-limb surgery**			

**Ref**	**Quality^a^** **EV PEDro**	**Analysis^b^** **Main short-term results**	**Effect sizes (absolute numbers)**

*Gauthier et al*[56]	2 | 4	Mann-Whitney *U*-test. No significant between-group differences^f^.	
*Hershko et al*[40]	2 | 5	Student's *t*-test & Chi-square test. The experimental groups improved significantly in PWB, whereas the control groups did not.	PWB = 1.22 (groups with Touch WB instruction) & 1.40 (groups with Partial WB instruction)
*Isakov*[41]	1 | 4	Student's *t*-test. Significant between-group difference in improvement in favor of experimental group.	FWB = **-** (mean improvement (SD) are given: 7.9 kg(5.3) *vs*. 7 kg(2.4)

References in italic represent the studies for which the added benefit of applying biofeedback could be evaluated.

Between-group, pre- to post-intervention effect sizes are given for outcomes with a significant group difference.

^a ^EV = score external validity (≤ 2), PEDro = PEDro score, i.e. score internal and statistical validity (≤ 10)

^b ^A group or pre- vs. post-test difference was regarded as significant if *p *< .05, unless indicated otherwise

^c ^Hatzitaki et al: subjects were divided into subgroups that practised weight-shifting in the anterior/posterior direction (E1) vs medio/lateral direction (E2)

^d ^Yavuzer et al: authors mentioned possible ceiling effect for pelvic obliquity during walking in the control group

^e ^Montoya et al: testing was performed at the beginning and end of each training session

^f ^Gauthier-Gagnon et al: authors reported high inter- and intra-subject variability in sway measures

The PEDro scores ranged from 2 to 7 (out of 10) with a median score of 5. In 6 RCTs [28,45,47,49,51,55], allocation of subjects into their respective groups was concealed. For 7 studies [26,28,41,44,46,56,57] it could not be determined that groups were similar at baseline regarding prognostic indicators. There was 1 study that adjusted for confounding factors in the analysis. In the study by Wolf et al [52], pre-intervention balance measures and subject characteristics were used as covariates to correct for baseline differences between groups. Blinding of subjects and therapists was not possible in any of the controlled trials. In only 3 articles [39,45,55] blinding of assessors to treatment allocation was reported. In 2 studies [44,55], post-intervention measurements were obtained from less than 85% of the subjects initially allocated to groups. In addition, for 2 other studies [46,52], it was not clear how many subjects performed the post-intervention tests. In the studies by Sihvonen et al [48] and Engardt [33], less than 85% of the subjects initially allocated to groups were available for follow-up testing. None of the 21 studies described an intention-to-treat analysis or specifically stated that all subjects received training or control conditions as allocated.

Remarks on validity and/or reliability of outcome assessments were made in 10 studies [28,40,41,45-47,49,51,55,56]. In particular, Isakov [41] conducted a separate study to establish the validity and reliability of a new, in-shoe, body-weight measuring device before applying it during an intervention. Bradley et al [28] also assessed the reliability of assessments in a pilot study prior to the intervention study. In addition, Sihvonen et al [49] estimated the reliability of dynamic balance tests by administrating the tests twice at baseline, with a 1 week interval. Furthermore, reliability was increased by using the best result out of 5 for further analysis. A similar method was used by Rose & Clark [46] to increase diagnostic tests reliability. In obtaining baseline measures, they conducted the tests twice on consecutive days and only used the scores of the second administration for the analysis.

### Effectiveness of biofeedback-based interventions

Table 4 shows the main short-term results of the 21 included intervention studies and the calculated effect sizes. In 13 studies [26,28,29,32,35,40,41,44,45,47,51,56,57], the added benefit of applying biofeedback for balance or mobility training could be evaluated (see table 3 for details on the comparison conditions). Nine of these studies demonstrated a significantly larger improvement in one or more outcomes for the biofeedback-based training (see table 4) and only 3 out of the 13 studies (i.e. Cheng et al [29], Engardt [32,33], Sackley & Lincoln [47]) conducted a follow-up test. None of the studies demonstrated significantly larger improvements for the training without biofeedback.

#### • Training balance with visual biofeedback in (frail) older adults

In 4 out of 4 studies, significant and moderate-to-large effects in favor of the training group compared to the control group, which did not receive exercise-based training, were found for force platform-based measures of postural sway during quiet standing. The same was found for weight-shifting during standing in 2 out of 2 studies. Long-term results for postural sway were evaluated in 2 studies. Significant effects in favor of the training group were reported at 4 weeks [49] or 4 months [52] after the intervention. In 2 out of 2 studies, a significant decrease in reaction time during quiet standing in favor of the training group was demonstrated. In addition, significant and small-to-moderate effects in favor of the training group were found for the Berg Balance Scale in 3 out of 3 studies.

#### • Training balance with visual biofeedback in older patients post-stroke

In 3 out of 3 studies, no significant differences in force platform-based measures of postural sway during quiet standing were found for biofeedback-based training versus similar training without biofeedback. However, in 2 out of 3 studies, significant effects in favor of the biofeedback-based training were found for weight-distribution during standing.

#### • Training gait with auditory (and visual) biofeedback in older patients post-stroke

In 3 out of 4 studies, the addition of auditory feedback on a specific aspect of gait during training led to significantly larger improvements for the trained aspect, i.e. step width, step length, or knee extension. However, in 2 out of 2 studies, no significant difference for training with or without auditory (and visual) feedback on the knee extension or muscle tone was found for the Rivermead Mobility Index or the gait subscale of the Motor Assessment Scale.

#### • Training sit-to-stand transfers with auditory or visual biofeedback in older patients post-stroke

In 2 out of 2 studies, the addition of feedback on weight-bearing during training led to significantly larger improvements, directly or 6 months after the intervention, for force platform-based measures of weight-distribution. The between-group, pre- to post-intervention effect sizes were moderate to large, i.e. 1.16 and .63 for rising; and 1.47 and .70 for sitting down.

#### • Training gait or balance with auditory biofeedback in older patients with lower-limb surgery

In 2 out of 2 studies, significantly larger improvements for weight-bearing were found after full or partial weight-bearing gait training with the addition of feedback on the weight that is born on the affected limb.

## Discussion

This review presents the first overview of available intervention studies on biofeedback-based training of balance or mobility tasks across older adults with different rehabilitation needs. The aims of the review were to evaluate the feasibility and the effectiveness of applying the biofeedback methods. After a broad literature search, 21 studies were identified that met the criteria for inclusion in evaluating the effectiveness. Since no selection criteria were applied regarding type of participants, besides the criterium of a mean age of 60 years or higher, the studies included different populations of mobility-impaired older adults as well as (frail) older adults without a specific medical condition.

Despite the systematic approach, some potential sources of bias, such as language and publication bias, may have influenced the results of the review. In addition, some relevant studies may have been overlooked since literature was searched for in common databases. Non-reporting of details in the identified articles contributed to a lack of a 100% agreement between raters in scoring methodological quality. A quantitative statistical pooling of data of different studies was not possible due to the large heterogeneity in study characteristics.

### Feasibility of biofeedback-based interventions in older adults

None of the available studies on biofeedback-based interventions for training balance or mobility tasks in older adults used a specified method, such as a patient satisfaction survey, to collect information on the practical applicability of the biofeedback method. Most studies did not specifically report on subjects that dropped-out of the intervention, participation rates and occurrence of adverse events due to the biofeedback-based intervention. In addition, subjects with co-morbidity, e.g. regarding musculoskeletal conditions, sensory and cognitive impairments, were largely excluded. Therefore, there is insufficient evidence on whether biofeedback methods can be successfully applied in older adults with disabling health conditions.

### Effectiveness of biofeedback-based interventions in older adults

Since no quantitative analysis was performed and since there were no large-scale RCTs among the included studies, definitive conclusions cannot be made. However, several relevant indications on the (added) effectiveness of biofeedback-based interventions were identified.

For training of balance tasks on a force platform or pressure sensors with display of visual feedback, indications for positive effects were identified in different groups of (frail) older adults without a specific medical condition. Next to training-specific effects, i.e. reduced postural sway and improved weight-shifting ability in standing, effects on the attentional demands in quiet standing and balance during functional activities as measured by the Berg Balance Scale were identified. Sustainability of improvements some time after the intervention was identified for postural sway. Whether the changes in mean score on the Berg Balance Scale for the biofeedback-based training groups, i.e. approximately 1 [42], 3.0 [46] and 3.4 points [49], reflect meaningful changes is not clear. Existing reports [59,60] mention different values concerning the change that is required to reflect a clinically significant improvement. Whether improvements in balance after the intervention are also reflected in a reduced incidence of falls remains unclear. Sihvonen et al [48] reported a significant effect of the visual feedback-based balance training compared to no training on recurrent falls (8% vs 55% of falls) during a 1-year follow-up period as well as a reduced risk of falling (risk ratio .398). However, in another well-designed RCT (by Wolf et al [53]) where improvements in balance and mobility were not evaluated, visual feedback-based balance training in 64 community-dwelling older adults did not lead to reduced fall incidents compared to no training. Since the 6 available studies did not compare the biofeedback-based training to other exercise-based training, it cannot be determined whether the improvements were specifically due to the biofeedback component.

Based on the available studies in older patients post-stroke, indications for larger improvements after training balance, gait or sit-to-stand transfers with biofeedback compared to similar training without biofeedback were identified for the aspects that were specifically trained with use of the biofeedback. The indications for larger improvement in weight-distribution and similar improvement in postural sway during standing for balance training with versus without visual biofeedback are in accordance with the reportings of meta-analyses in general populations of patients post-stroke by van Peppen et al [18] and Barclay-Goddard et al [17]. The addition of biofeedback during gait training does not seem to lead to larger benefits for mobility functioning, since no difference between training with and without biofeedback was reported for the Rivermead Mobility Index in one study and for the gait subscale of the Motor Assessment Scale in another study. Also, for gait training in older patients with lower-limb surgery, an indication for larger improvement with the addition of auditory biofeedback was identified for the trained aspect, i.e. the weight on the affected leg.

### Future directions

Current studies do not yet provide clear indications regarding the long-term additional benefit of applying biofeedback in interventions for balance and mobility training in older populations. In addition, it is difficult to determine how much additional improvement is obtained due to the biofeedback method, since differences in the performed analyses and the reporting of results between studies prevent the calculation of effect sizes that can be compared across studies. The available studies provide limited information on whether biofeedback-based training of balance and/or mobility has effect on disability and functioning. The model of disability of The International Classification of Functioning, Disability and Health (ICF) by the World Health Organization (WHO) demonstrates that outcomes need to be evaluated at different domains and levels in order to describe changes in functioning. In the present systematic review, indications for improvement were identified primarily for outcomes at the activity domain on a capacity level, i.e. outcomes that quantify the highest possible ability to execute a task in a standardized environment. It is not clear whether any improvements in laboratory-based measures of balance or mobility are reflected in a larger reduction of falls and in a better ability to execute mobility tasks in the daily life environment.

Further studies are needed that evaluate the added effectiveness as well as the feasibility of applying biofeedback-based training methods in geriatric practice and that include other older populations than patients post-stroke or with lower-limb surgery. Since the research quality of most of the current studies is moderate, further studies should include large number of participants, apply concealment of allocation of subjects into their respective groups and adjust for confounding factors in the statistical analysis. Besides optimizing the research quality, the reporting of studies should be optimized by showing whether groups were similar at baseline regarding prognostic indicators as well as by mentioning details on blinding of assessors, validity and reliability of the outcome assessments, and on the number of subjects that completed the intervention and assessments. To be able to implement results to geriatric practice, future studies should focus on biofeedback systems that can be applied in the every-day clinical setting and allow for practicing of tasks that resemble every-day life challenges and can be applied during a prolonged period of time. Recent progress in technology for wearable, wireless systems to monitor human motion [61] can facilitate the development of biofeedback systems that can be used in every-day settings. Within the European Commision-funded project SENSACTION-AAL (FP6), an audio-biofeedback system based on a wireless tri-axial accelerometer worn at the lower back has been developed for use in the home environment.

## Conclusions

Due to a lack of systematic evaluations of feasibility aspects in the available intervention studies up to 2010, there are no clear indications yet regarding the feasibility of applying biofeedback-based methods for training balance or mobility tasks in geriatric practice. Concerning the effectiveness, relevant indications for improvement on training-specific aspects of balance or mobility exist. However, further appropriate intervention studies are needed to be able to make definitive statements regarding the (long-term) added effectiveness, particularly on measures of functioning in older adults with different rehabilitation needs.

## List of abbreviations used

ADL: Activities of Daily Living; BBS: Berg Balance Scale; BF: BioFeedback; BI: Barthel Index; C: Control group; CB&M: Community Balance and Mobility; COG: Center Of Gravity; COP: Center Of Pressure; E: Experimental group; EMG: ElectroMyoGraphic; 100%LOS: 100% Limits Of Stability test; MAS: Motor Assessment Scale; PED: Physiotherapy Evidence Database; P/FWB: Partial/Full Weight-Bearing; rANOVA: repeated measures ANalysis Of Variance; (R)CT: (Randomized) Controlled Trial; RMA: Rivermead Motor Assessment; RMI: Rivermead Mobility Index; RT: Reaction Time; SOT: Sensory Organization Test; TUG: Timed Up & Go test; vGRF: vertical Ground Reaction Force; conv.: conventional; max.: maximum; sign.: significance.

## Competing interests

The authors declare that they have no competing interests.

## Authors' contributions

AZ participated as a first reviewer in the design of the study and the data collection, performed the analyses and drafted the manuscript. MM participated in the design of the review, and participated as a second reviewer in the study selection and quality assessment and helped in the data interpretation and revising the manuscript. WZ conceived the study, and participated in its design, participated as a third reviewer and helped to draft the manuscript. LC helped in the design of the review and revising the manuscript. All authors read and approved the final manuscript.
